# Conservatively Managed, Displaced Scaphoid Fracture in a Young, Female Collegiate Basketball Player

**DOI:** 10.7759/cureus.9793

**Published:** 2020-08-16

**Authors:** Samantha Braun, Ashley Yelinek

**Affiliations:** 1 Physical Medicine and Rehabilitation, Sports Medicine, West Virginia School of Osteopathic Medicine, Clarksburg, USA; 2 Sports Medicine, United Hospital Center, Clarksburg, USA

**Keywords:** scaphoid fracture, wrist fracture, fall on outstretched hand injury, scaphoid fracture treatment, diagnosis of scaphoid fracutre

## Abstract

Fractures of the scaphoid bone occur in 60-70% of wrist bone fractures. It most often occurs after a fall onto outstretched hand injury. Many times, the diagnosis of a scaphoid fracture is missed due to the unique anatomy and positioning of this carpal bone. Treatment options vary depending on the type of fracture and degree of displacement. We present the case of a 19-year-old female patient. She reported to the urgent care facility after a fall on an outstretched hand while playing basketball. She was diagnosed with a wrist sprain. After having continued pain she sought care at the sports medicine clinic where she was diagnosed with a displaced scaphoid fracture. She was managed conservatively with thumb-spica casting and later adjuvant therapy treatments with low-intensity pulsed ultrasound (LIPUS) technology.

## Introduction

Fractures of the carpal bones are a very common injury, with 60-70% of fractures occurring in the scaphoid bone [1]. Duckworth et al. reported an overall incidence of 29 per 100,000 population (95% CI, 25-34) [2]. The most common mechanism of injury is a fall from standing height onto an outstretched hand. It is also closely linked to sports including basketball, cycling, and skateboarding [2]. The scaphoid bone has unique anatomy including a “boat-like” shape, retrograde vascular supply, and an oblique positioning. It serves as the link between the proximal and distal carpal rows. There are many classification systems for scaphoid fractures based on fracture location, plane orientation, and stability/displacement. Of these systems, one of the most used is the Herbert or Modified Herbert System which is based on fracture stability [3]. Management of scaphoid fractures varies depending on the location, displacement, stability, and individual patient demographics. The distinctive function, anatomy, and location of the scaphoid lend itself to unique challenges and controversy regarding diagnosing, treating, and rehabilitating injuries of the scaphoid. Scaphoid carpal bone fractures are common, and their diagnosis and management are widely surrounded by debate. The following case is presented as an example of a conservatively managed, displaced scaphoid fracture in a collegiate athlete.

## Case presentation

We report a case of a healthy, 19-year-old right-handed, female, collegiate basketball player who originally presented to an urgent care facility in November 2019. She fell onto outstretched hands while playing basketball. She reported immediate right wrist pain after the fall, but was able to continue playing. X-rays were taken and she was diagnosed with a right wrist sprain. The patient reported the initial X-rays to be negative. Further records from the outside facility were unable to be obtained. She then presented to us (Orthopedic and Sports Medicine Clinic) in January 2020 with continued right wrist pain. She described the pain at the base of her right thumb, extending into her proximal wrist. The pain was characterized as constant, sharp, and stabbing, with mild to moderate pain at its worst. It was worse with any attempted movement of both the wrist and thumb. She had worn a wrist brace without improvement in symptoms. She applied ice compresses and used oral over the counter pain medication. The patient had no previous trauma, pain, or abnormal history with her right wrist. She reported no medical history, surgical history, or current medication. She has continued to play basketball since the original injury.

Vitals included: height 1.75 m (5’ 8.9”), weight 73.5 kg (162 lbs.), BMI 23.99 kg/m^2^. Physical exam was benign except for the following musculoskeletal findings: right wrist with mild swelling, but normal joint alignment and no atrophy. The patient had tenderness over the scaphoid and anatomical snuffbox with no other bony tenderness. She had increased pain upon palpation, near full range of motion (ROM). Strength was 5/5 in all planes, but with increased pain. Radial pulses were +2/4. Our differential diagnosis included: right carpal scaphoid fracture, wrist sprain, contusion of carpal soft tissue, De Quervain’s Tenosynovitis, and contusion of carpal bone without fracture.

**Figure 1:**
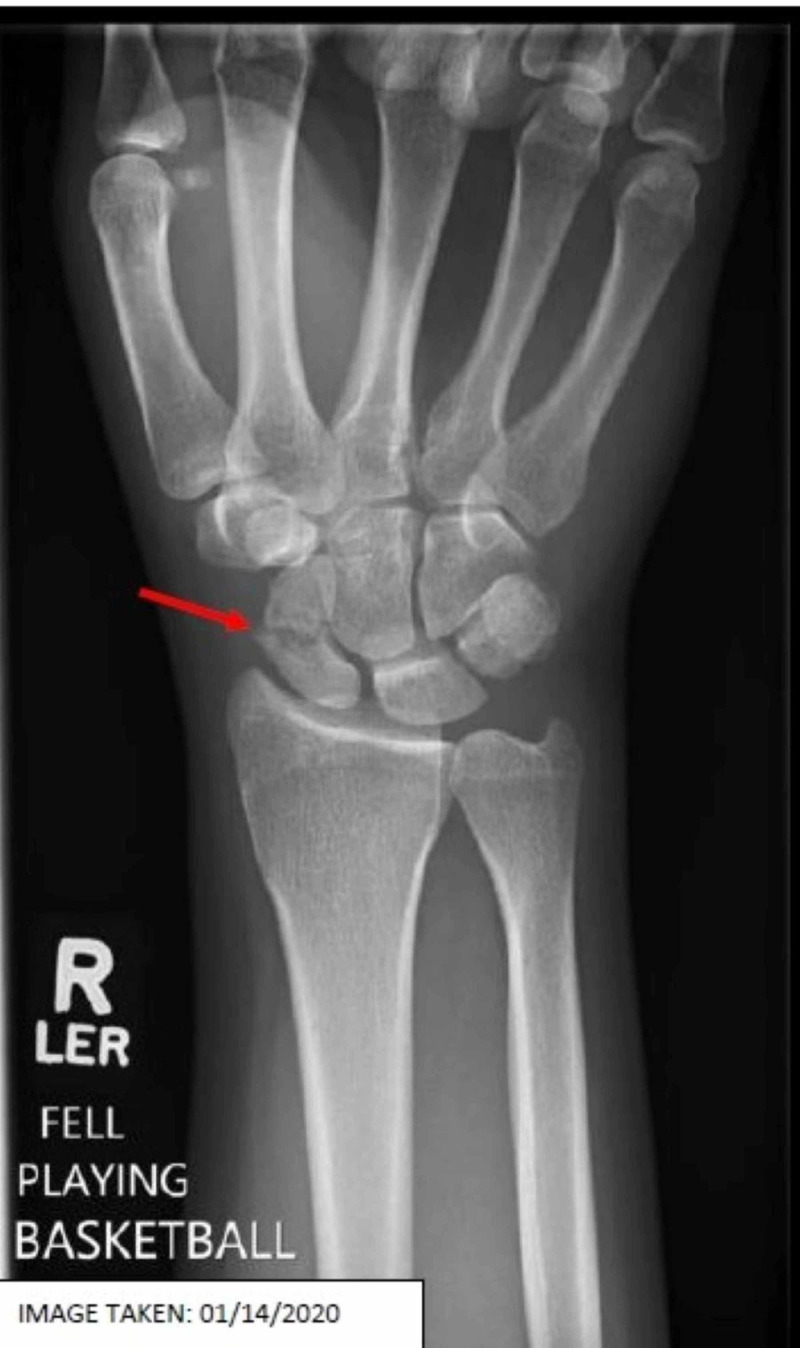
X-ray revealed scaphoid waist fracture with mild displacement, but overall good alignment, minimal callus, and incomplete healing (Red arrow)

An X-ray in January 2020 was obtained and read with an impression suggesting a scaphoid waist fracture with mild displacement, but overall good alignment, minimal callus, and incomplete healing (Figure 1). The patient’s final diagnosis was a closed displaced fracture of the scaphoid waist of the right wrist with delayed healing.

Clinical course

Once the diagnosis was made the patient was placed in a below-the-elbow thumb spica cast. In addition to casting, it was decided due to non-union and continued pain that a bone stimulator would be used as adjuvant therapy. The bone stimulator generated low-intensity pulsed ultrasound (LIPUS) technology. Daily LIPUS treatments were prescribed for 20 minutes for one month or until appropriate bone growth was achieved as evident via X-ray.

**Figure 2:**
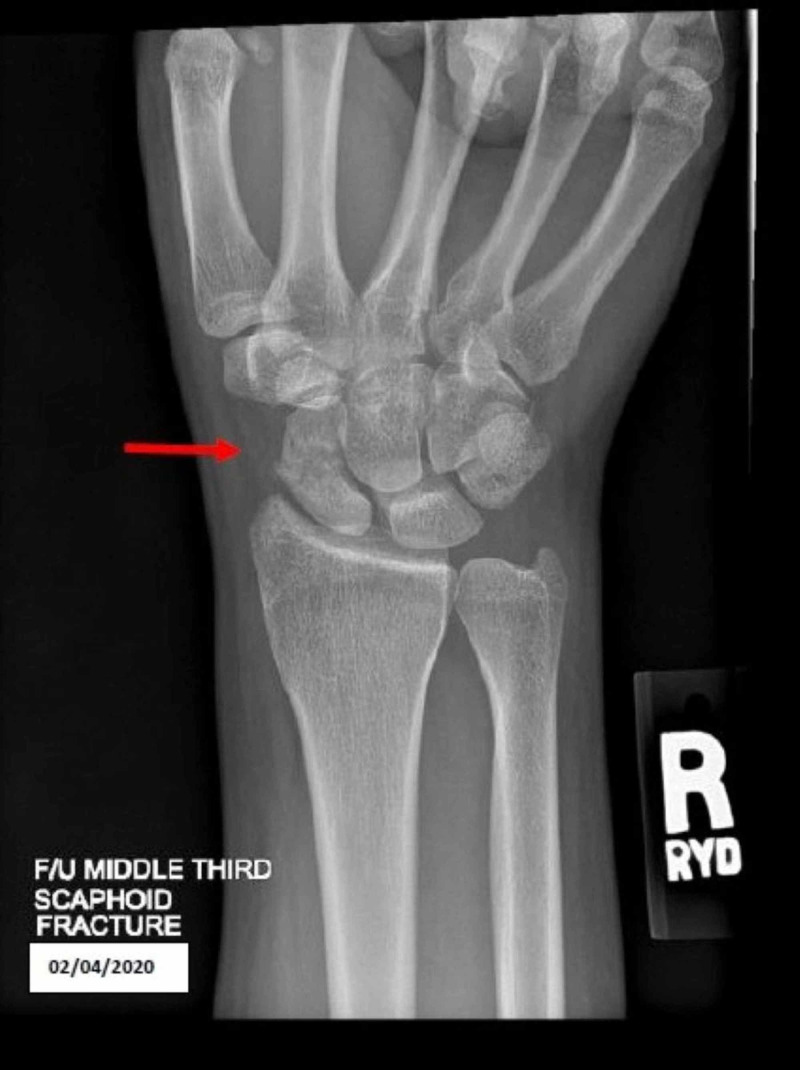
X-ray revealed stable alignment of healing fracture of the scaphoid with an interval increase in osseous bridging compared to prior (Red arrow)

The patient returned for a follow-up exam and X-rays three weeks post initial visit. She had been compliant with her cast and did undergo bone stimulator treatments for one week before the appointment. She denied having current pain. On physical exam, the right wrist was without edema or effusion. She had mild, but improving tenderness over the scaphoid and anatomic snuffbox and near full ROM with mild stiffness. Muscle strength was intact at 5/5, but with increased pain. An X-ray revealed stable alignment of healing fracture of the scaphoid with an interval increase in osseous bridging compared to prior (Figure 2). The thumb-spica cast was continued along with LIPUS bone stimulator treatments. Restrictions included no right upper extremity weight-bearing, heavy lifting, or contact sports. The patient will return in three weeks for an X-ray.

**Figure 3:**
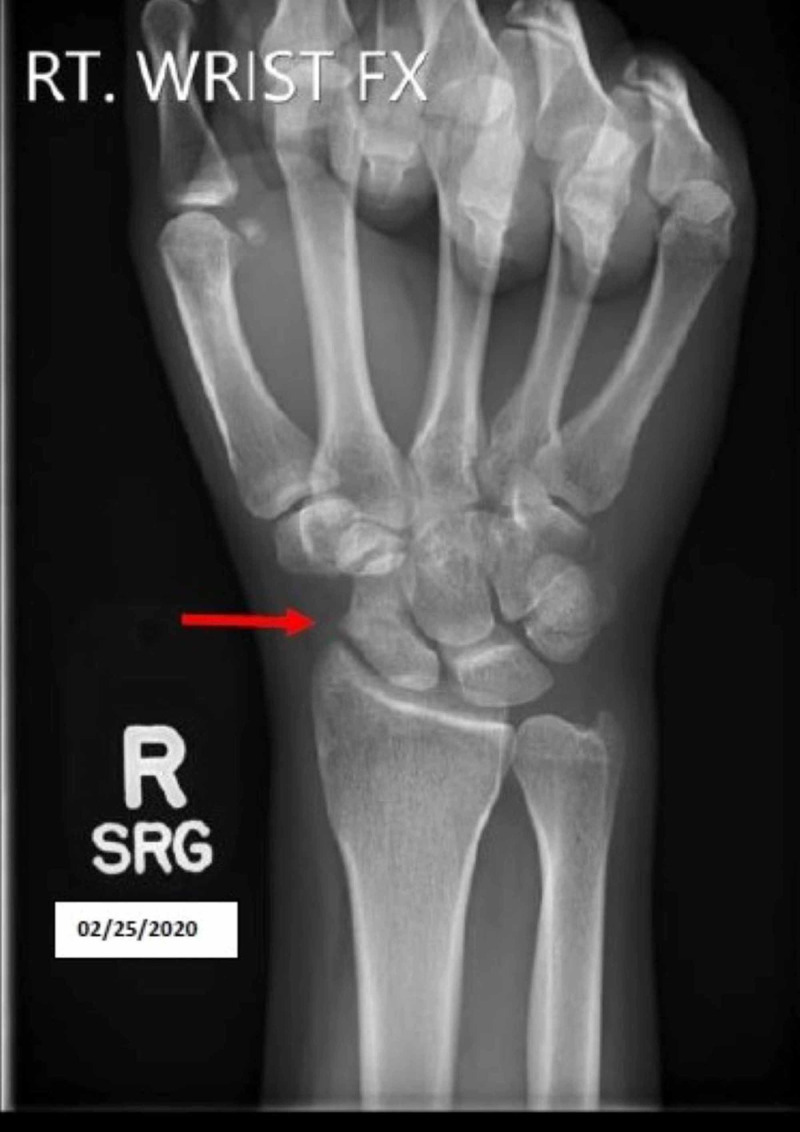
X-ray revealed increased healing of scaphoid waist fracture with mild displacement, overall good alignment, and continued callus formation with still incomplete healing (Red arrow)

Upon the patient's return, three months from the original injury, she reported no pain. On physical exam, she was able to make “okay” and “thumbs up” signs. Her ROM was intact with muscle strength 5/5. An X-ray revealed increased healing of scaphoid waist fracture with mild displacement, overall good alignment, and continued callus formation with still incomplete healing (Figure 3). Her thumb spica cast was removed and replaced with a thumb spica brace. She was advised to continue LIPUS bone stimulator treatments. At this time, she was given rehabilitation to perform at home and was also released to perform physical therapy and rehabilitation with her collegiate athletic trainers.

Because of the COVID-19 pandemic, this patient did not return to our clinic and may have returned to her home out-of-state since her academic studies were moved online and her collegiate basketball season and spring training were canceled.

## Discussion

Scaphoid fractures are the most frequent carpal fracture [4]. It is the second most common fractured bone in the upper extremity after the distal radius [5]. Using the Herbert Classification, the most reported fracture was a displaced waist fracture (B2). The incidence of scaphoid fractures was reported as 29 per 100,000 of the population [2]. It should be noted, there are variable reports of incidence, most often contributed to the retrograde analysis, low capture rates, small study population sizes, and the difficulty surrounding imagining and interpretation of the fracture.

Anatomically, the scaphoid has an irregular shape often described as a boat-like. Eighty percent of the bone is covered in cartilage, limiting the vascular flow which is supplied by the radial artery via mostly retrograde flow [6]. Its anatomical position serves as the link between the proximal and distal carpal rows. The scaphoid long axis is tilted volarly and radially concerning the longitudinal aspect of the limb [5].

Most often a scaphoid fracture will present after a fall on an outstretched hand with the wrist in radial deviation [6]. Duckworth et al. reported low energy falls from standing height were most common followed by contact sports such as basketball, football, and skateboarding. The median age of injury was 27 years [2].

A physical exam provides a useful tool when ruling out scaphoid fractures. Sensitivity is 100% when pain is reported and elicited when applying pressure on the anatomic snuffbox or the scaphoid tubercle or when applying axial pressure on the first metacarpal bone [6].

Based on physical exam and clinical suspicion, imaging should be obtained. There are several imaging modalities used in the assessment of scaphoid fractures including conventional radiographs, computed tomography (CT), magnetic resonance imaging (MRI), and bone scintigraphy but are individually limited due to cost and radiation exposure. All imaging modalities are vulnerable to interpretation error and image quality. Literature suggests first obtaining anterior-posterior, lateral, and oblique views in addition to a scaphoid view. This view is described as the wrist partially supinated posteroanterior with ulnar deviation. However, due to the oblique positioning, scaphoid fractures are often missed or misinterpreted based on stability. Bernard et al. reported that standard wrist radiographs had a 78% sensitivity for correctly identifying displaced fractures and even lower specificity (68%) for correctly excluding fracture displacement. CT scans allow for improved assessment of cortical involvement. CT scans, along with or in combination with radiographs, improved the reliability of the diagnosis of a scaphoid fracture displacement [7].

Treatment of the fracture should aim to attain functional recovery while avoiding nonunion or avascular necrosis. The treatment of scaphoid fractures significantly lacks clear recommendations on the best treatment modalities based on fracture classifications. Research comparing conservative versus nonconservative management of scaphoid fractures is convoluted and is limited due to retrograde analysis and small study population sizes. For nondisplaced (stable) fractures, most can be immobilized in a cast for 8-12 weeks. Nonetheless, there are no distinctly clear guidelines as to which route of immobilization is best. The scaphoid is difficult to immobilize, nearly every motion of the hand, wrist, and forearm creates movement of the bone [6]. Physicians are often faced with deciding the cast duration, length, involvement of the thumb, and wrist flexion or extension. In a meta-analysis comparing conservative (nonsurgical) methods for acute scaphoid fracture, Doornberg et al. found that there was no statistical difference in time to union, pain, grip strength, immobilization time, and range of motion, except for wrist extension. Wrist extension was significantly more limited in patients immobilized in flexion [8].

Physicians may consider adjuvant therapy with low-intensity pulsed ultrasound (LIPUS) or pulsed electromagnetic fields as bone stimulations as treatments. Since the scaphoid presents such a unique anatomy, there is use for LIPUS therapy. Mayr et al. concluded that low-intensity ultrasound is successful in accelerating the healing of scaphoid fractures. It should be noted this study only had 30 randomized cases [9].

Displaced (unstable) fractures are most often managed surgically due to the anatomical and vascular challenges of the scaphoid bone. Operative techniques allow for a minimally invasive approach to stabilization of the bone and earlier return to activity. In a meta-analysis comparing surgical versus conservative treatments for acute displaced or minimally displaced scaphoid fractures, there was a statistically significant difference in the mean functional scores, return to work time, and grip strength of the surgical group. However, there was no statistically significant difference in the range of motion and complications (malunion, nonunion, osteoarthritis), the need for further surgical treatments, avascular necrosis, infection, and procedure failure.

In addition to the lack of clear evidence for the treatment of scaphoid fractures in the general population, there is often debate surrounding surgical versus non-surgical treatment based on patient hand dominance and certain demographics such as employment or athletic involvement. Most often, athletes with a scaphoid fracture of their dominant hand are treated with surgical options for various reasons including return to play. This can be supported by a meta-analysis performed by Al-Ajmi et al. which revealed that the return to work time was significantly lower (p < 0.05) among patients treated surgically [10]. In the case presented, it was decided to try non-operative treatments first with the thought that if she did not achieve adequate healing on X-ray, she would advance to surgical treatments to ensure complete healing of the fracture. Like many practicing physicians, this decision was made based on previous cases encountered in their practices as well as advice gathered from colleagues.

## Conclusions

Due to the location, function, and anatomy of the scaphoid bone, a multimodal approach to diagnosis and treatment may result in the best patient outcomes. A displaced scaphoid fracture that does not heal properly can result in prolonged pain, limited work function, decreased economic factors for the patient, and possibly in the death of the bone, which in itself can lead to a chronic arthritic condition. The diagnosis and treatment of a displaced fracture are difficult and often variable depending on the clinical presentation and imaging modality used. A fracture in which the scaphoid is displaced or has a simple fracture occurring near the end of the bone should require conclusive diagnosis via X-ray including views in anterior-posterior, lateral and oblique views and a scaphoid view. If after X-ray, the diagnosis is inconclusive, otherwise other imaging modalities such as CT should be used if available and a scaphoid fracture suspected. Bracing and casting should be left to the treating physician's choice as studies reported no significant difference in pain, function, and healing. Many should consider adjuvant therapy with low-intensity pulsed ultrasound (LIPUS) or pulsed electromagnetic fields as bone stimulations as treatments. Additional research conducted with larger sample populations, standardized imaging, and treatment algorithms would be very beneficial to advance our knowledge on this very common fracture.
